# Health science students’ preparedness for climate change: a scoping review on knowledge, attitudes, and practices

**DOI:** 10.1186/s12909-024-05629-2

**Published:** 2024-06-11

**Authors:** Fabricio Ccami-Bernal, Fernanda Barriga-Chambi, Carlos Quispe-Vicuña, Daniel Fernandez-Guzman, Rodolfo Arredondo-Nontol, Miriam Arredondo-Nontol, David Rojas-Rueda

**Affiliations:** 1 Universidad Nacional de San Agustín de Arequipa, Arequipa, Perú Peru; 2 Sociedad Científica San Fernando, Universidad Nacional Mayor de San Marcos, Lima, Perú; 3 Carrera de Medicina Humana, Universidad Científica del Sur, Panamericana Sur km 19, Lima, Perú; 4 Escuela Profesional de Medicina Humana de la Universidad Nacional de Tumbes, Tumbes, Perú; 5 Hospital Carlos Alberto Cortez Jiménez Essalud Tumbes, Tumbes, Perú; 6 Department of Environmental and Radiological Health Sciences, Colorado State University, Fort Collins, CO USA; 7 Colorado School of Public Health, Colorado State University, 1601 Campus Delivery, Fort Collins, CO 80523 USA

**Keywords:** Climate change, Health occupations students, Attitude, Knowledge, Practice, Global health

## Abstract

**Introduction:**

Climate change (CC) is a global public health issue, and the role of health professionals in addressing its impact is crucial. However, to what extent health professionals are prepared to deal with CC-related health problems is unclear. We aimed to evaluate the knowledge, attitudes, and practices of health students about the CC.

**Methods:**

We conducted a scoping review through systematic searches in PubMed, Scopus, Web of Science, Proquest, and EBSCO. We included original scientific research with no language or time restrictions. Two authors independently reviewed and decided on the eligibility of the studies, then performed data extraction.

**Results:**

21 studies were included, with a total of 9205 undergraduate nursing, medical, pharmacy, and public health students mainly. Most health science students (> 75%) recognized human activities as the main cause of CC. However, they perceived a lack of knowledge on how to address CC. Moreover, we found inadequate coverage or limited development of CC in related curricula that may contribute to incomplete learning or low confidence in the theoretical and practical concepts of students.

**Conclusion:**

The findings of our scoping review suggest that while health sciences students possess a general understanding of CC, there is a significant gap in their knowledge regarding its specific health impacts. To address this gap, there is a need for targeted education and training for future health care professionals that emphasizes the health effects of CC.

**Supplementary Information:**

The online version contains supplementary material available at 10.1186/s12909-024-05629-2.

## Introduction

The increasing use of fossil fuels and the release of greenhouse gases have led to an increase in the temperature of the environment and variations in climatic phenomena. Thus, climate change (CC) is one of the main public health problems with global reach [1] as it plays an important role in several environmental determinants of health, such as air and water pollution, as well as food shortages, and droughts, among others [2]. In addition, CC favors the development of communicable and non-communicable diseases, either directly or indirectly [3]. Such is the impact of CC that, in the last 20 years, it has been attributed to more than 5 million deaths worldwide [4]. By 2030, approximately 250,000 deaths are expected, generating annual public health expenditures of nearly US$4 billion [5].

All these threats highlight the need for multilevel strategies to prevent future fatal events and the loss of country economies [6]. Health professionals have a key role in addressing CC threats to health [7, 8] because they are directly confronted with its impact, as is the case of the increase in cases of infectious diseases each year, or the increased mortality of patients with chronic diseases during a heat wave [9]. In the field of health sciences, medical education on climate change seems variable, as on the one hand there is evidence of incomplete knowledge despite the fact that students would like to learn more and on the other hand it is reported that most of the teaching given comes from individual initiatives rather than from a formal status in their syllabus [10]. Therefore, public health measures at different levels and by health institutions to mitigate the effects of CC are necessary to address this problem [11]. To this end, it is essential to teach future health professionals about CC and its impacts on health to better face these challenges and consequences [12, 13].

Many students may become future public health decision-makers, and thus, their training should ensure knowledge about CC. However, it has been reported that about 13% of students receive an environmental health education within the curricula of their professional schools [14]. On the other hand, the curricular structure of the courses taught is likely not the most adequate to face the challenges that arise or come as a result of CC [15, 16], indicating that health professionals will not be prepared to face the challenges that CC poses [17]. This scenario could lead to serious public health consequences, as health professionals will play a key role in addressing the challenges and health threats caused by CC [17].

Among the main solutions is the need to promote public awareness of CC and advocate for the population’s health by communicating opportunities and threats to public policymakers [8]. Despite this, it is not entirely clear to what extent health professionals are prepared to act and respond adequately to the health impacts of CC. In that sense, a university education would be an appropriate starting point to prepare health professionals, as higher education is one of the pillars of sustainable development in the world [18, 19] and moreover, It has been reported that the concern and awareness of medical and nursing students about causes of CC may be influenced by the knowledge acquired, especially in its causes [20, 21]. . It should also be added that although there are previous reviews that evaluate education on the environment and climate change in medical student populations (human medicine, nursing, etc.) [10, 22, 23], these only perform a superficial analysis without highlighting the knowledge, attitude or practice of each student in each study. Therefore, this scoping review assessed knowledge, attitudes, mitigation practices against CC of health students and the students’ perspectives regarding the incroporation of CC in the academic curriculum. These findings will provide an updated overview of the problem and serve as a basis for stimulating and implementing topics related to CC in curricular plans.

## Methods

A scoping review was conducted following the guidelines of the 2018 Preferred Reporting Items for Systematic Analysis and Meta-Analysis for Scoping Reviews (PRISMA-ScR) extension [24], and the methodology described by the Joanna Briggs Institute [25]. The review protocol has been publicly disclosed and is accessible online [26]. The primary objective of this study was to assess the knowledge, attitudes, and practices related to climate change among students in health sciences. As a secondary aim, we examined the students’ perspectives regarding the incrorporation of this topic into their curricula was evaluated.

### Eligibility criteria

In this scoping review, we included original scientific research published in scientific journals, without language or time restriction, that assessed knowledge, attitudes, practices, or related attributes of health science students about CC. We included students of health sciences from professional schools or formal educational programs in medicine, nursing, dentistry, pharmacy, and psychology.

### Literature search

The following databases were searched: (1) PubMed, (2) Scopus, (3) Web of Science (Core collection), (4) Proquest (Health & Medical Collection, Public Health Database, Education Database, Environmental Science Database, Psychology Database, Nursing, and Allied database) and (5) EBSCO (Dentistry & Oral Sciences Source, GreenFILE, Psychology, and Behavioral Sciences Collection). The search was conducted on May 4, 2022. The complete search strategy for each database can be found in Supplementary Material 1.

### Study selection

**Fig. 1:**
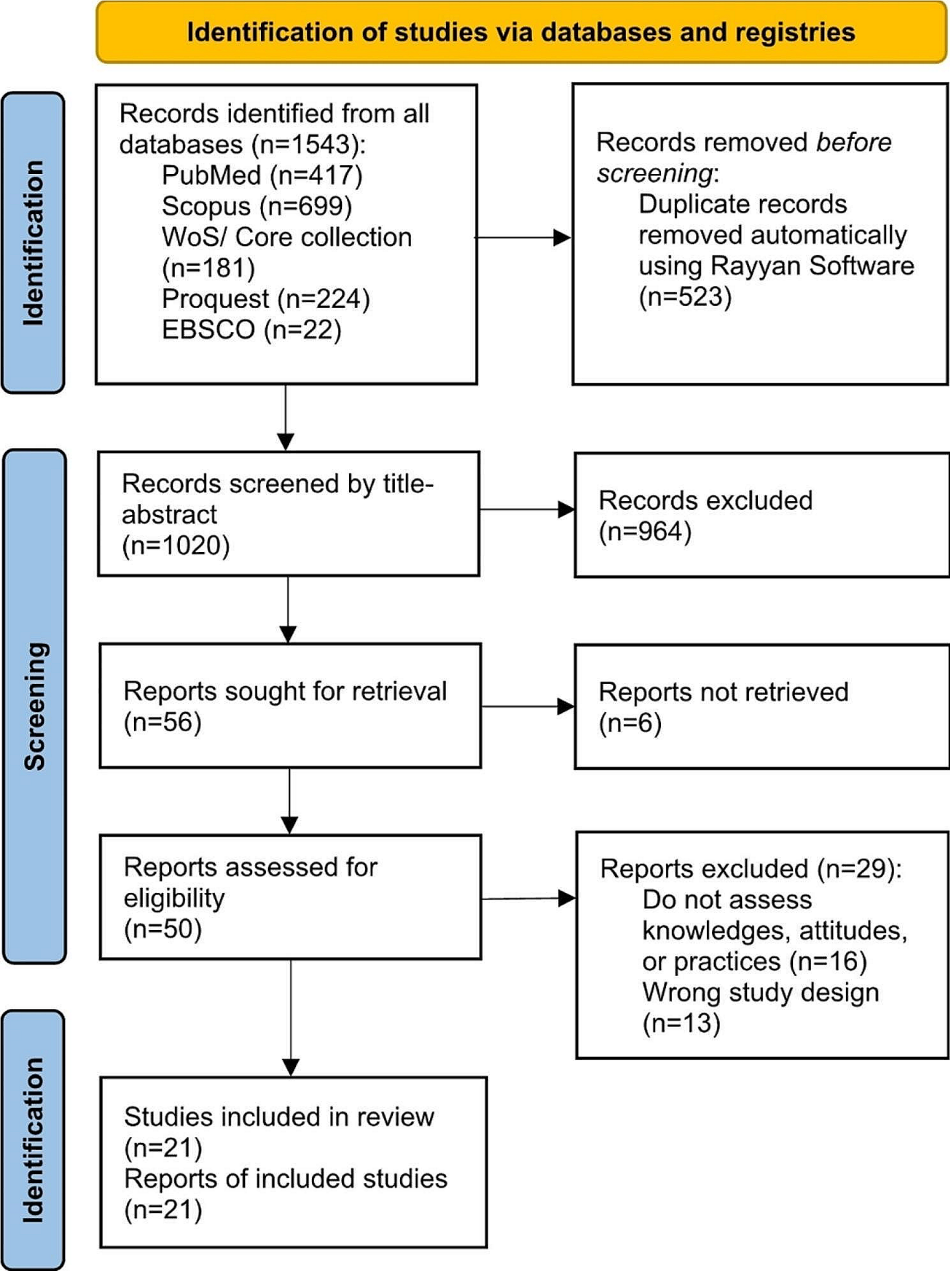
Flow diagram summarizing the process of literature search and selection

Articles from the databases were imported into Rayyan Software, and duplicates were manually removed. Subsequently, two authors (C.Q.V., D.F.G.) independently screened by title and abstract to sift potentially includable articles. Then, two authors (M.A.N., R.A.N.) independently screened full-text studies for compliance with the selection criteria. Discrepancies were resolved at a meeting with a third author (F.C.B. or D.F.G.). The screening and selection flowchart are shown in Fig. 1.

### Data extraction

We designed a Microsoft Excel sheet to extract the data. This process was performed independently by two authors (F.C.B., M.B.C.). We extracted the following variables from the studies included: name of the first author, date of publication, region, the characteristics of the study (type and focus of the study, the instruments or methods used to assess the knowledge, attitudes, practices (KAPs), and their validity), the gross national income of the country of the study population as established by the World Bank [27], the characteristics of the students (type of students, year of study, and sample size). KAPs on the following items were extracted: Knowledge (causes of CC, effects on the environment and human health, perception of the level of knowledge and sources of information), attitudes according to the definition used in the primary studies (importance of CC and role as a health professional) and mitigation practices against CC (eco-friendly practices by the health system and at home) and the inclusion of CC in the academic curriculum. In the case of longitudinal studies, data were extracted from the baseline data.

### Synthesis of results

A scoping rather than a systematic review was proposed because it is necessary to identify and map the evidence on the topic addressed, the types of studies, and the concepts used to examine how research in this field is conducted [28]. The synthesis of the results was narrative, applying a qualitative thematic analysis approach to categorize and present the key themes in our data. The contents of the studies were synthesized in an evidence map, identifying common themes, and synthesizing the logical link between them, as well as the research gaps identified.

## Results

**Fig. 2:**
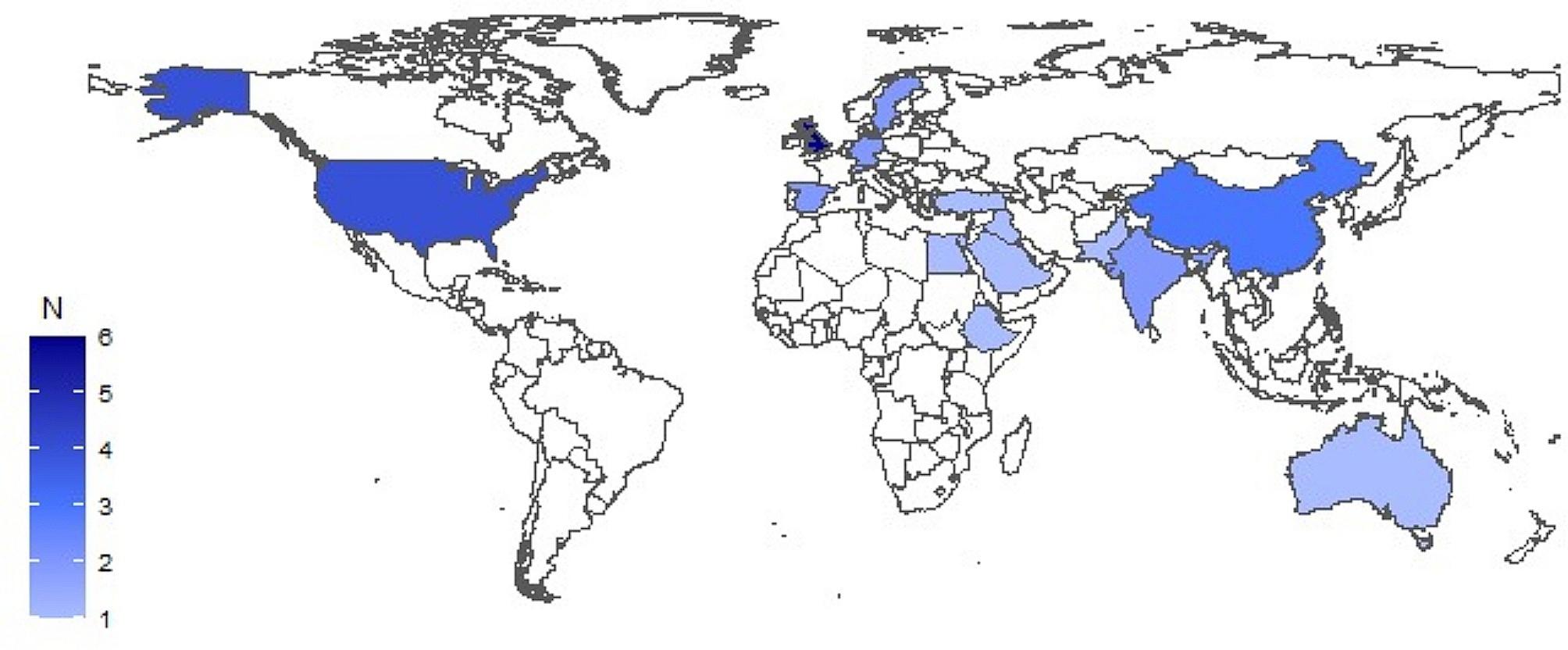
Geographic representation of the countries studied in the studies included (*n* = 35)

Of a total of 1020 studies identified, 21 including 9205 students were finally included [13, 21, 29–47] (Fig. 1). Sixteen (76.2%) were cross-sectional, and four (19.0%) were qualitative studies. Of the 35 countries studied, Europe, the Middle East, and Asia were the most studied regions with 14 (40%), 10 (28.5%), and 5 (14.3%) respectively, the remaining 6 studies (17.2) belong to the USA, Africa, and Australia (Fig. 2), with high-income countries being the most studied. Fourteen studies (66.7%) included nursing students, ten (47.6%) medical students, two (9.5%) pharmacy students, two (9.5%) public health students, and four (19%) included other health students (physician assistants, medical laboratory technology, environmental health, health officers, psychiatry nursing, dentistry, midwifery). The students were mostly fourth-year (10 studies), third-year (10 studies), first-year (9 studies), and second-year (7 studies). 50% of the articles used instruments developed by the authors, and the questionnaire most commonly used was the Sustainability Attitudes in Nursing Survey (SANS) questionnaire (33.3%) (Table 1).

**Table 1:** Characteristics of the studies included (*n* = 21) with 35 countries: Europe: 14, Middle East: 10, Asia: 5, USA: 4, Africa: 1, Australia: 1

Author and publication year	Population				Methods		
	Target students	Students year	Sample size	Country	Study design	Study type	Instrument
Álvarez-Nieto (2022) [29]	Nursing	1st	846	UK, Spain, Germany, Sweden, Australia	Cross-sectional	Quantitative	SANS
Anåker (2022) [30]	Nursing	3rd	12	Sweden	Cross-sectional	Qualitative	Interviews
Bugaj (2021) [31]	Medical	6th	65	Germany	Cross-sectional	Quantitative	Self-developed
Kliger (2021) [13]	Medical	1st	36	USA	Longitudinal	Quantitative	Environmental Health in Med School (EHMS) survey
Jamal (2020) [32]	Medical	5th	333	Pakistan	Cross-sectional	Quantitative	Self-developed
Ryan (2020) [33]	Medical, nursing, physician	1st to 5th	280	USA	Cross-sectional	Quantitative	Self-developed
Yang (2018) [21]	Medical, nursing, public health	3rd and 4th	1387	China	Cross-sectional	Quantitative	Self-developed
Liao (2019) [34]	Medical	3rd and 4th	1387	China	Cross-sectional	Quantitative	Self-developed
Gruenberg (2017) [35]	Pharmacy	3rd	115	USA	Longitudinal	Quantitative	Self-developed
Richardson (2015) [36]	Nursing	1st	916	UK, Germany, Spain, Switzerland	Cross-sectional	Quantitative	SANS
Richardson (2016) [37]	Nursing	2nd	57	UK	Longitudinal	Quantitative	SANS
Nigatu (2014) [38]	Medical, nursing, pharmacy, public health and others*	1st to 4th	306	Ethiopia	Cross-sectional	Quantitative	Self-developed
Pandve (2011) [39]	Medical	1st to 3rd	250	India	Cross-sectional	Quantitative	Self-developed
Richardson (2019) [40]	Nursing, midwifery	3rd	246 − 145	UK	Longitudinal	Quantitative	SANS
Linton (2020) [41]	Nursing	4th	91	USA	Longitudinal	Quantitative	SANS
Felicilda-Reynaldo (2017) [42]	Nursing	2nd to 4th	1059	Egypt, Iraq, Palestinian Territory, and Saudi Arabia	Cross-sectional	Quantitative	New Ecological Paradigm (NEP) scale
Regan (2012) [43]	Medical	2nd to 4th	24	UK	Cross-sectional	Qualitative	Focus group
Parashar (2013) [44]	Medical, nursing, dentist	1st	400	India	Cross-sectional	Quantitative	Self-developed
Ergin (2021) [45]	Nursing	4th	164	Turkey	Cross-sectional	Mixed	Global Warming Questionnaire (GWQ)
Cruz (2018) [46]	Nursing	2nd to 4th	1059	Egypt, Iraq, Palestinian Territory, and Saudi Arabia	Cross-sectional	Quantitative	SANS
Chen (2020) [47]	Nursing	1st	101	UK, China	Cross-sectional	Mixed	SANS and interviews

*UK*: United Kingdom, *USA*: United States of America, *SANS* Sustainability Attitudes in Nursing Survey questionnaire

*Medical Laboratory Technology, Environmental Health, Health Officer, and Midwifery

**Fig. 3:**
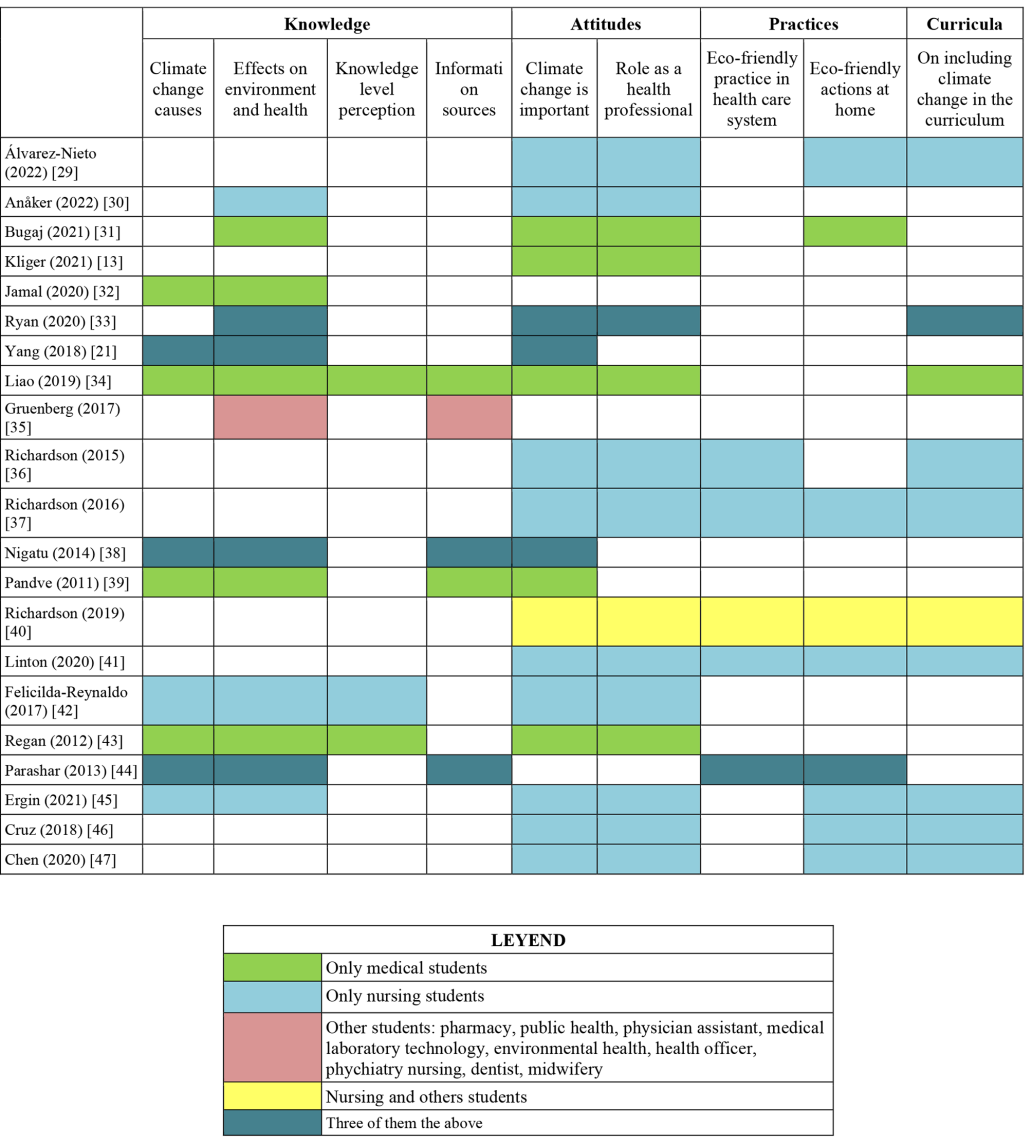
Evidence map

Table 2 and the evidence map (Fig. 3) summarize the KAPs assessed. Attitudes, practices, and opinions regarding the curriculum were studied more by nursing students, while knowledge was assessed more by medical students. Of the 11 studies that included medical students, 10 assessed knowledge, 8 attitudes, and 2 practices. Of the 14 studies that included nursing students, 6 assessed knowledge, 14 attitudes, and 9 practices. Of the 6 that evaluated students from other careers (pharmacy, public health, physician assistant, medical laboratory technology, environmental health, health officer, psychiatry nursing, dentist, midwifery), 5 evaluated knowledge, 4 attitudes, and 2 practices.

**Table 2:** Key findings and recommendations

Key findings	
•	Attitudes, practices, and opinions regarding the curriculum were studied more by nursing students, while knowledge was assessed more by medical students.
•	More than 75% of students in the included studies identify • human activities as the cause of climate change.
•	The most frequently identified effects of CC on health included issues such as food and water scarcity, alterations in air quality, and heat shocks. However, students expressed a perceived lack of information or knowledge essential for addressing the impacts of CC on health adequately.
•	The predominant portion of students in the included studies expressed concern about the health effects of CC and recognized their responsibility to conserve resources and prevent contamination within their professional practice.
•	Medical students exhibit lower belief and personal interest in addressing climate change in healthcare compared to nursing students, and are less inclined to play an educational role.
•	Students supported reducing unnecessary materials in healthcare and valued eco-friendly measures, including transparent environmental practices and optimization of resources
•	Students support the inclusion of climate change (CC) in the curriculum, preferring its integration into clinically focused courses emphasizing practical skills.
Recommendations	
•	Curriculum developers in the health science fields should prioritize including CC and its effect on health in their teaching, with a practical approach and a focus on necessary skills.
•	It is necessary to study this issue in health sciences students from low to middle-income countries and regions where it has not yet been studied, especially those more vulnerable to climate change.
•	The promotion of the study of this topic is recommended among students, such as those in the fields of medicine or psychology, given the limited existing literature on the subject despite its significance.
•	Qualitative or mixed-methods studies related to the knowledge, attitudes, and practices of health sciences students regarding climate change (CC) could adopt a more holistic approach in this field, warranting promotion. Additionally, longitudinal studies assessing the effects of interventions, such as curriculum inclusion, would be valuable.

### Knowledge

Thirteen studies reported knowledge of CC [21, 30–35, 38, 39, 42–45]. Eight assessed the knowledge of the causes of CC. In one study, the majority of nursing students (65.4%) considered CC to be a balance between natural and human causes [42], unlike the rest of the studies [21, 32, 34, 39, 43–45] in which more than 75% of the students identified the contribution of human activities (industrial and vehicular pollution, deforestation, unsustainable consumption of resources, CO_2_ emissions) as a cause of CC. Only one study assessed knowledge about pollution produced by the area of health, with less than half of the students correctly identifying that “10% of gas emissions come from the health sector”, and with less knowledge among medical students [33].

The 13 studies assessed knowledge about the effects of CC on the environment and health. The effects on the environment most frequently identified were lack of food and water [30, 31, 45], changes in air quality and heat shocks [27], and an increase in poverty and migration [30]. Regarding health effects, more than 90% of students identified the interruption in the delivery of health services [21, 33, 34] and diseases related to air quality [33, 34, 45]. More than 60% of students linked skin cancer to increased ultraviolet radiation [32, 45]. Other effects identified to a lesser extent were vector-related infectious diseases [38, 39, 43], malnutrition, water quality [38], mental health conditions [33, 38], and cold-related illness [21].

In four studies, most students reported feeling that they do not have the information or knowledge necessary to appropriately address the impact of CC on health [13, 34, 42, 43] and one was associated with a lack of adequate teacher orientation and/or inadequate practical training one study described a lack of adequate teacher orientation and/or inadequate practical training [34]. In addition, when CC information sources were consulted, the internet was the most popular source in a study conducted by medical and nursing students (95%) [34], in contrast to other health students among whom only 45% used internet as a main source of information [34]. The second most used source of information was the television and radio [34, 38, 40].

### Attitudes

Eighteen studies assessed attitudes toward CC [13, 21, 29–31, 33, 34, 36–43, 45–47]. In the studies that used the SANS questionnaire for nursing students; two reported that students mildly agreed with the statement. “Climate change is an important issue for nursing” [36, 37], four stated that they somewhat agreed [29, 40, 41, 47] and another study reported that 55% agreed with the statement to some degree [46]. Similarly, the majority (> 80%) of students consider CC to be a serious, important, and current problem [21, 34, 38]. In qualitative studies, nursing students have a pessimistic view of the future concerning CC [30], and medical students believe the health impact will be massive [43].

Students believe the topic is important and can help their patients, but medical students believe it to a lesser extent than nursing students (60.5% vs. 81.1%) [33]. Similarly, medical students were more likely to say that CC is not an area of personal interest to them (57.4% vs. 39.6%) [33]. Although students consider that they should have an educational role in their patients and the public about the impacts of CC [31], medical students consider it to a lesser extent (69.8% vs. 86.8%) [33]. Moreover, when asked if they feel prepared for CC, 2 out of 3 medical students believe they are not prepared in to deal with CC [13].

Regarding the responsibilities that health professionals should assume in CC, approximately 90% are concerned about the health effects of CC and recognize that they are responsible for conserving resources and preventing contamination within their professional practice [33, 34]. Similarly, in the qualitative studies, students stated that health professionals should be role models for patients and society regarding CC [30, 43, 45]. However, this professional responsibility was less assumed in the case of medical students [31] and about 80% of health science students consider pollution prevention and natural resource conservation outside their responsibility [33, 38].

### Practices

Ten studies reported on practices related to CC [29, 31, 36, 37, 40, 41, 44–47]. Regarding eco-friendly practices within the health care system, 95% of students agreed on reducing unnecessary material used in health care [33]. More than 80% of students recognized the importance of promoting measures such as transparency of the environmental footprint of the health care industry and optimization of supplies, procedures, and medical devices [33].

Of the studies on nursing students, in two, the mean areed with the statement: “They apply sustainability principles in nursing practice” [40, 41], and in two studies the meanwas neutral [36, 37]. On eco-friendly practices in the home, the average mildly agreed [37, 46, 47] and somewhat agree [29, 40, 41] with the statement: “they apply sustainability principles at home.” 77% of nursing students choose eco-friendly products [45]. Similarly, in the case of medical students, about 75% perform eco-friendly actions such as avoiding the consumption of plastic [31] or energy saving [44], and, to a lesser extent (30%), recycling or use of alternative energies [44].

### Curriculum

Ten studies analyzed the opinions of including CC in the curriculum [29, 33, 34, 36, 37, 40, 41, 45–47]. The mean of the nursing students agreed to some degree with including CC and sustainability to the nursing curriculum [29, 40, 41, 46, 47], in contrast to the rest of the studies in which the mean was neutral [36, 37]. This is similar to other studies on medical and nursing students, among whom 90% consider the need to include CC in the curriculum [33, 34]. However, one out of three studied mentioned that there might not be time to learn about this topic because of the pre-existing academic load [33].

With respect to how to include CC in the curriculum, students preferred that the topics should be addressed during classes in a stand-alone or existing course [33, 34, 45] focused on clinical knowledge and skills related to CC in a practical and clinically integrated manner [34]. Therefore, they stated that the knowledge should be reinforced in a clinical setting [21]; this opinion was higher among nursing students and physician assistants (71.7%) than in medical students (57.4%) [33].

## Discussion

Our study found that most health science students (> 75%) recognized human activities as the main cause of CC. However, while they were aware of the health consequences of CC, they perceived a lack of knowledge on how to address them. Moreover, we found inadequate coverage or limited development of CC in related curricula, which may contribute to the incomplete learning or low confidence in the theoretical and practical concepts of students on the subject. Despite this, researchers are increasingly interested in understanding the KAP. of health care professionals, especially those who provide direct patient care and are involved in community activities such as nursing. We found that nursing students were more predominant in the studies we evaluated than medical or other health professionals.

Although a university education is an essential pillar for building student knowledge and skills, learning scenarios (courses, workshops, seminars, etc.) on CC and health are either not included in the related curricula or developed only to a limited extent [16, 48, 49]. Nonetheless, student initiatives are leading the way to addressing the issue in the educational setting [50], which provides an opportunity to propose programs that can strengthen education and equip health professionals to face the threats of CC in health [51]. Students agreed that CC is an important problem for their professions and future patients, but medical students expressed less concern compared to nursing or other professionals. This concern and awareness of the relevance of CC were also observed in other university students [52] and health professionals who have already graduated [53, 54]. Conversely, physicians in India have expressed more interest in learning about and addressing climate change and its effects on health than other professionals [55]. This could be due to the practical setting, in which graduating physicians better perceive the knowledge gap needed to address this problem with their patients [56].

Regarding practices, most students recognized the importance of complying with eco-friendly practices within the health care system and in their homes. Promoting such behaviors is crucial, as the health sector is one of the primary greenhouse gas emitters [57]. Health professionals have enormous potential to shift quickly from being consumers of sectors that degrade the environment to drivers of the green revolution, as they have an ethical mandate not to harm [57].

Finally, most students believe that CC teaching should be included in their curricula, with a practical, clinical approach that does not add to the academic load of other courses. A study evaluating the teaching of CC to medical students using didactic and experiential elements showed a significant increase in their self-reported sense of readiness to discuss the importance of CC with their patients [13]. Therefore, efforts should be continued to adequately include CC in the curriculum. Similarly, international student organizations, such as the International Federation of Medical Students’ Associations, advocate a multi-pronged approach to incorporate the teaching of CC in the medical curriculum and across the entire spectrum of the curricula of health professions [58].

This scoping review has certain limitations that need to be acknowledged. Firstly, only studies in English were included, which may have resulted in overlooking some relevant studies. In addition, we did not formally assess the methodological quality of the studies included, which might affect the reliability of the results reported. Secondly, this review followed the methodological recommendations of JBI [25] and PRISMA [24], which may have limited the scope of the review. Thirdly, the studies included primarily represented students from middle- to high-income countries, which restricts the generalizability of the findings to middle- to low-income countries. Caution should be exercised when generalizing the findings to all students, as this scoping review did not undertake quantitative synthesis. Nevertheless, this review is the first to synthesize and provide an overview of the literature in this study area, which is a significant strength of the study.

## Conclusion

The findings of our scoping review suggest that while health sciences students possess a general understanding of climate change (CC), there is a significant gap in their knowledge regarding the specific health impacts of CC. To address this gap, there is a need for targeted education and training of future health care professionals that emphasizes the health effects of CC. Such training should also focus on practical, applicable approaches to managing and mitigating the impacts of CC on patient health. Additionally, our review highlights the strong desire of students for CC education to be integrated into their curricula in a more experiential, hands-on way. Ultimately, the results of our study underscore the importance of addressing these gaps in knowledge and training among health science students to equip them to better meet the challenges of CC in their future careers.

### Electronic supplementary material

Below is the link to the electronic supplementary material.

Supplementary Material 1

## Data Availability

All data generated or analysed during this study are included in this published article.
