# Supplementary material for: Health science students’ preparedness for climate change: a scoping review on knowledge, attitudes, and practices
Source: BMC Med Educ. 2024 Jun 11;24:648. doi: 10.1186/s12909-024-05629-2 (PMC11167912; doi:10.1186/s12909-024-05629-2)
Supplement: Supplementary file 1 — Supplementary Material 1 [file 12909_2024_5629_MOESM1_ESM.docx]

**Supplementary Material 1. Search strategy.**

**Date of search:** 03/10/22

**Filters:** None

| Databases | Set | Search terms | Items found |
| --- | --- | --- | --- |
| PUBMED | 1 | "Climate change*" OR "Change, Climate" OR "Changes, Climate" OR "Global Warming" OR "Warming, Global" OR "global warm*" OR "sea level rise" OR "Rising Sea Level" OR "Rising Sea Levels" OR "Sea Level, Rising" OR "Sea Levels, Rising" OR "Sea-Level Rise" OR "greenhouse effect" OR "Effect, Greenhouse" OR weather OR "environmental change" OR "Environmental Indicators" OR "Environmental Indicator" OR "Indicator, Environmental" OR "Indicators, Environmental" OR "Environmental Biomarkers" OR "Biomarkers, Environmental" OR "Environmental Biomarker" OR "Bioindicator" OR "Bioindicators" OR "Biological Indicator" OR "Biological Indicators" OR "Indicator, Biological" OR "Indicators, Biological" OR "climate disaster" OR "Climate emergency" OR "climate disruption" OR "climate variability" OR "climatic variability" OR "carbon emission" OR “climatic change” | 654.199 |
|  | 2 | "Students, Health Occupations"[MeSH Major topic] "Students, Health Occupations"[tiab] OR "Health Occupations Students"[tiab] OR "Students, medical" [MeSH Major topic] OR "Medical Students"[tiab] OR "Student, Medical"[tiab] OR "Medical Student"[tiab] OR "Students, dental" [MeSH Major topic] OR "Dental Students"[tiab] OR "Student, Dental"[tiab] OR "Dental Student"[tiab] OR "Students, nursing" [MeSH Major topic] OR "Pupil Nurses"[tiab] OR "Student, Nursing"[tiab] OR "Nurse, Pupil"[tiab] OR "Pupil Nurse"[tiab] OR "Nursing Student"[tiab] OR "Nursing Students"[tiab] OR "Students, pharmacy" [MeSH Major topic] OR "Pharmacy Students"[tiab] OR "Student, Pharmacy"[tiab] OR "Pharmacy Student"[tiab] OR "Students, premedical" [MeSH Major topic] OR "Premedical Students"[tiab] OR "Student, Premedical"[tiab] OR "Premedical Student"[tiab] OR "preclinical student*"[tiab] OR "student physician*"[tiab] OR "Students, public health" [MeSH Major topic] OR "Health Students, Public"[tiab] OR "Public Health Student"[tiab] OR "Student, Public Health"[tiab] OR "Public Health Students"[tiab] OR "Education, Medical"[MeSH Major topic] OR "Education, Nursing"[MeSH Major topic] OR "Education, dental"[MeSH Major topic] OR "Education, pharmacy"[MeSH Major topic] OR "student doctor*"[tiab] OR "clinical student*"[tiab] OR "trainee doctor"[tiab] OR "public health student*"[tiab] OR "medical school"[tiab] OR "medical education"[tiab] OR "medical undergraduate"[tiab] OR "undergraduate medic*"[tiab] OR "dental student*"[tiab] OR "dental school"[tiab] OR "dental education"[tiab] OR "dental undergraduate"[tiab] OR "dentistry student*"[tiab] OR "nursing student*"[tiab] OR "nursing school"[tiab] OR "nursing education"[tiab] OR "nursing undergraduate"[tiab] OR "pharmacy student*"[tiab] OR "pharmacy school"[tiab] OR "pharmacy education"[tiab] OR "pharmacy undergraduate"[tiab] OR "psychology student*"[tiab] OR "psychology school"[tiab] OR "psychology education"[tiab] OR "psychology undergraduate"[tiab] | 318.020 |
|  | 3 | S1 AND S2 | 417 |
| SCOPUS | 1 | TITLE-ABS-KEY("climate change*” OR “climatic change*” OR "global warm*" OR "sea level rise" OR "rising sea level*" OR "sea-level rise" OR "greenhouse effect*" OR weather OR "environmental change" OR "environmental indicator*" OR "Environmental Biomarker*" OR "Bioindicator*" OR "Biological Indicator*" OR "climate disaster*" OR "climate emergency" OR "climate disruption" OR "climate variability" OR "climatic variability" OR "carbon emission") | 827.852 |
|  | 2 | TITLE-ABS-KEY("Health occupations students" OR "medical student*” OR "dental student*" OR "nursing student*" OR "pupil nurses" OR "pharmacy student*" OR "premedical student*"OR “preclinical student*" OR "student physician*" OR "public health student*" OR "medical education" OR "nursing education" OR " dental education" OR "pharmacy education" OR "student doctor*" OR "clinical student*" OR "trainee doctor" OR "medical school" OR "medical undergraduate" OR "undergraduate medic*" OR "dental school" OR "dental undergraduate*" OR "dentistry student*" OR "nursing school" OR "nursing undergraduate" OR "pharmacy school" OR "pharmacy undergraduate" OR "psychology student*" OR "psychology school" OR "psychology education" OR "psychology undergraduate") | 534.584 |
|  | 3 | S1 AND S2 | 699 |
| Web of Science (Core collection) | 1 | TS**=**("climate change*” OR “climatic change*” OR "global warm*" OR "sea level rise" OR "rising sea level*" OR "sea-level rise" OR "greenhouse effect*" OR weather OR "environmental change" OR "environmental indicator*" OR "Environmental Biomarker*" OR "Bioindicator*" OR "Biological Indicator*" OR "climate disaster*" OR "climate emergency" OR "climate disruption" OR "climate variability" OR "climatic variability" OR "carbon emission") | 524294 |
|  | 2 | TS=("Health occupations students" OR "medical student*” OR "dental student*" OR "nursing student*" OR "pupil nurses" OR "pharmacy student*" OR "premedical student*"OR “preclinical student*" OR "student physician*" OR "public health student*" OR "medical education" OR "nursing education" OR " dental education" OR "pharmacy education" OR "student doctor*" OR "clinical student*" OR "trainee doctor" OR "medical school" OR "medical undergraduate" OR "undergraduate medic*" OR "dental school" OR "dental undergraduate*" OR "dentistry student*" OR "nursing school" OR "nursing undergraduate" OR "pharmacy school" OR "pharmacy undergraduate" OR "psychology student*" OR "psychology school" OR "psychology education" OR "psychology undergraduate") | 106479 |
|  | 3 | S1 AND S2 | 181 |
| Proquest (Health & Medical Collection,  Public Health Database,  Education Database,‎  Environmental Science Database,‎  Psychology Database, Nursing and Allied database)‎ | 1 | AB(("climate change" OR "climate changed" OR "climate changes") OR ("climatic change" OR "climatic changes") OR ("global warming") OR "sea level rise" OR "rising sea level*" OR "sea-level rise" OR ("greenhouse effect" OR "greenhouse effects") OR weather OR "environmental change" OR ("environmental indicator" OR "environmental indicators") OR "Environmental Biomarker*" OR "Bioindicator*" OR ("biological indicators") OR "climate disaster*" OR "climate emergency" OR "climate disruption" OR "climate variability" OR "climatic variability" OR "carbon emission") | 269242 |
|  | 2 | AB("Health occupations students" OR ("medical student" OR "medical students") OR ("dental student" OR "dental students") OR ("nursing student" OR "nursing students") OR "pupil nurses" OR ("pharmacy student" OR "pharmacy students") OR "premedical student*"OR “preclinical student*" OR "student physician*" OR "public health student*" OR "medical education" OR "nursing education" OR " dental education" OR "pharmacy education" OR ("student doctor" OR "student doctors") OR "clinical student*" OR "trainee doctor" OR "medical school" OR "medical undergraduate" OR ("undergraduate medical") OR "dental school" OR "dental undergraduate*" OR "dentistry student*" OR "nursing school" OR "nursing undergraduate" OR "pharmacy school" OR "pharmacy undergraduate" OR ("psychology student" OR "psychology students") OR "psychology school" OR "psychology education" OR "psychology undergraduate") | 107122 |
|  | 3 | S1 AND S2 | 224 |
| EBSCO (Dentistry & Oral Sciences Source,  GreenFILE and  Psychology and Behavioral Sciences Collection) | 1 | ("Climate change*" OR "Change, Climate" OR "Changes, Climate" OR "Global Warming" OR "Warming, Global" OR "global warm*" OR "sea level rise" OR "Rising Sea Level" OR "Rising Sea Levels" OR "Sea Level, Rising" OR "Sea Levels, Rising" OR "Sea-Level Rise" OR "greenhouse effect" OR "Effect, Greenhouse" OR weather OR "environmental change" OR "Environmental Indicators" OR "Environmental Indicator" OR "Indicator, Environmental" OR "Indicators, Environmental" OR "Environmental Biomarkers" OR "Biomarkers, Environmental" OR "Environmental Biomarker" OR "Bioindicator" OR "Bioindicators" OR "Biological Indicator" OR "Biological Indicators" OR "Indicator, Biological" OR "Indicators, Biological" OR "climate disaster" OR "Climate emergency" OR "climate disruption" OR "climate variability" OR "climatic variability" OR "carbon emission" OR "climatic change") | 127087 |
|  | 2 | ("Students, Health Occupations" OR "Students, Health Occupations" OR AB "Students, Health Occupations") OR (TI "Health Occupations Students" OR AB "Health Occupations Students") OR "Students, medical" (MM +) OR (TI "Medical Students" OR AB "Medical Students") OR (TI "Student, Medical" OR AB "Student, Medical") OR (TI "Medical Student" OR AB "Medical Student") OR "Students, dental" (MM +) OR (TI "Dental Students" OR AB "Dental Students") OR (TI "Student, Dental" OR AB "Student, Dental") OR (TI "Dental Student" OR AB "Dental Student") OR "Students, nursing" (MM +) OR (TI "Pupil Nurses" OR AB "Pupil Nurses") OR (TI "Student, Nursing" OR AB "Student, Nursing") OR (TI "Nurse, Pupil" OR AB "Nurse, Pupil") OR (TI "Pupil Nurse" OR AB "Pupil Nurse") OR (TI "Nursing Student" OR AB "Nursing Student") OR (TI "Nursing Students" OR AB "Nursing Students") OR "Students, pharmacy" (MM +) OR (TI "Pharmacy Students" OR AB "Pharmacy Students") OR (TI "Student, Pharmacy" OR AB "Student, Pharmacy") OR (TI "Pharmacy Student" OR AB "Pharmacy Student") OR "Students, premedical" (MM +) OR (TI "Premedical Students" OR AB "Premedical Students") OR (TI "Student, Premedical" OR AB "Student, Premedical") OR (TI "Premedical Student" OR AB "Premedical Student") OR (TI "preclinical student*" OR AB "preclinical student*") OR (TI "student physician*" OR AB "student physician*") OR "Students, public health" (MM +) OR (TI "Health Students, Public" OR AB "Health Students, Public") OR (TI "Public Health Student" OR AB "Public Health Student") OR (TI "Student, Public Health" OR AB "Student, Public Health") OR (TI "Public Health Students" OR AB "Public Health Students") OR (MM "Education, Medical"+) OR (MM "Education, Nursing"+) OR (MM "Education, dental"+) OR (MM "Education, pharmacy"+) OR (TI "student doctor*" OR AB "student doctor*") OR (TI "clinical student*" OR AB "clinical student*") OR (TI "trainee doctor" OR AB "trainee doctor") OR (TI "public health student*" OR AB "public health student*") OR (TI "medical school" OR AB "medical school") OR (TI "medical education" OR AB "medical education") OR (TI "medical undergraduate" OR AB "medical undergraduate") OR (TI "undergraduate medic*" OR AB "undergraduate medic*") OR (TI "dental student*" OR AB "dental student*") OR (TI "dental school" OR AB "dental school") OR (TI "dental education" OR AB "dental education") OR (TI "dental undergraduate" OR AB "dental undergraduate") OR (TI "dentistry student*" OR AB "dentistry student*") OR (TI "nursing student*" OR AB "nursing student*") OR (TI "nursing school" OR AB "nursing school") OR (TI "nursing education" OR AB "nursing education") OR (TI "nursing undergraduate" OR AB "nursing undergraduate") OR (TI "pharmacy student*" OR AB "pharmacy student*") OR (TI "pharmacy school" OR AB "pharmacy school") OR (TI "pharmacy education" OR AB "pharmacy education") OR (TI "pharmacy undergraduate" OR AB "pharmacy undergraduate") OR (TI "psychology student*" OR AB "psychology student*") OR (TI "psychology school" OR AB "psychology school") OR (TI "psychology education" OR AB "psychology education") OR (TI "psychology undergraduate" OR AB "psychology undergraduate") | 21636 |
|  | 3 | S1 AND S2 | 22 |
